# Consumers’ Perceptions about Edible Insects’ Nutritional Value and Health Effects: Study Involving 14 Countries

**DOI:** 10.3390/ani14111631

**Published:** 2024-05-30

**Authors:** Raquel P. F. Guiné, Sofia G. Florença, Cristina A. Costa, Paula M. R. Correia, Nada M. Boustani, Irina Matran, Krešimir Jakšić, Cristina Chuck-Hernández, Elena Bartkiene, Ilija Djekic, Maria Papageorgiou, Leticia G. Arias, Malgorzata Korzeniowska, Maša Černelič-Bizjak, Dace Klava, Vanessa Ferreira, Emel Damarli, Manuela Ferreira

**Affiliations:** 1 CERNAS-IPV Research Centre, Polytechnic University of Viseu, 3504-510 Viseu, Portugal; sofiaflorenca@outlook.com (S.G.F.); amarocosta@sc.ipv.pt (C.A.C.); paulacorreia@esav.ipv.pt (P.M.R.C.); 2 Faculty of Business and Administration, Saint Joseph University, Beirut 1104 2020, Lebanon; nada.mallahboustany@usj.edu.lb; 3 Department of Community Nutrition and Food Safety, George Emil Palade University of Medicine, Pharmacy, Science, and Technology of Targu Mures, 540139 Targu Mures, Romania; irina.matran@umfst.ro; 4 Department of Psychology, University of Zadar, 23000 Zadar, Croatia; kjaksic@unizd.hr; 5 Tecnologico de Monterrey, The Institute for Obesity Research, Monterrey 64849, Mexico; cristina.chuck@tec.mx; 6 Department of Food Safety and Quality, Lithuanian University of Health Sciences, LT-47181 Kaunas, Lithuania; elena.bartkiene@lsmuni.lt; 7 Department of Food Safety and Quality Management, Faculty of Agriculture, University of Belgrade, 11000 Belgrade, Serbia; idjekic@agrif.bg.ac.rs; 8 Department of Food Science and Technology, International Hellenic University, 57001 Thessaloniki, Greece; mariapapage@food.teithe.gr; 9 BALAT Research Group, Faculty of Veterinary Medicine, University of León, 24071 León, Spain; lgona@unileon.es; 10 Faculty of Food Science, Wroclaw University of Environmental and Life Sciences, 51-630 Wrocław, Poland; malgorzata.korzeniowska@upwr.edu.pl; 11 Department of Nutritional Counseling—Dietetics, Faculty of Health Science, University of Primorska, 6320 Izola, Slovenia; masa.cernelic@fvz.upr.si; 12 Faculty of Food Technology, Latvia University of Life Sciences and Technologies, LV-3001 Jelgava, Latvia; dace.klava@llu.lv; 13 Department of Nutrition, School of Nursing, UFMG—Federal University of Minas Gerais, Belo Horizonte 30130-100, Brazil; vanessa.nutr@gmail.com; 14 Altıparmak Food Coop Research & Development Center, Çekmeköy, Istanbul 34782, Turkey; 15 Health Sciences Research Unit: Nursing (UICISA: E), Polytechnic University of Viseu, 3504-510 Viseu, Portugal; mmcferreira@gmail.com

**Keywords:** knowledge, edible insects, factor analysis, nutritional value, health effect

## Abstract

**Simple Summary:**

Climate change is one of the drivers of change towards sustainable food systems food security. Therefore, food security is a priority all around the world and across different sectors of society. Edible insects are recommended as a sustainable source of food of animal origin, but their acceptance is very diverse across cultures and countries. Therefore, our work investigated the perceptions of consumers about edible insects in 14 countries. We concluded that depending on origin, the level of knowledge is different, which is a starting point to design more focused campaigns to promote EIs, not only in non-insect-eating countries, but also in insect-eating countries. Better knowledge about the health effects of EIs and their nutritive value is a driver of change.

**Abstract:**

Insects have been consumed for time immemorial in many regions of the globe. However, in other parts, they are not traditionally eaten. Because they are a more sustainable source of animal protein and provide valuable nutrients as well as bioactive compounds with beneficial effects on the human body, their consumption is encouraged. Knowledge can serve as a tool for better acceptance of insects as food. In this context, the present work investigated the knowledge about the nutritional value and health effects of edible insects in different countries. Data were collected by employing a questionnaire survey translated into the different languages of all participating countries and were treated using statistical tools. A total of 7222 responses were obtained. The results indicated that for many issues, the participants manifested a neutral opinion (neither agree nor disagree), but the participants who manifested agreement/disagreement were generally well informed. They were also able to identify untrue facts and answer accordingly by disagreeing. Factor analysis showed four groups of questions: nutritive value, negative perception and risks, safety and benefits of insects and contamination and harmful components. Finally, significant differences were observed according to the sociodemographic variables studies (sex, age, education, living environment and country), with age and country being the most influential of the sociodemographic factors on knowledge. Therefore, increasing knowledge is envisaged as an essential factor in augmenting the recognition of edible insects as a nutritional food, presenting health benefits apart from being a more sustainable source of animal protein when compared with beef or pork meats.

## 1. Introduction

The consumption of insects as food has been a long tradition for some populations worldwide, but they are not at all part of the cultural gastronomic heritage of other regions. The practice of eating insects, known as entomophagy, has a contribution to the feeding habits of some populations, having both nutritional and health roles. The practice of eating insects is incorporated into the traditional gastronomic culture in many societies, particularly in tropical and sub-tropical countries in Asia, Africa and Latin America [1]. In contrast, in Western countries, most consumers are not familiar with edible insects (EIs), and some have not even tried them [2]. Nowadays, most of the insects available on the market are farmed and then processed to powder, being incorporated into other foods more familiar to Western consumers [3]. Sogari et al. [4] provided an overview of entomophagy at present in both insect-eating communities and non-insect-eating ones. Their study included participants from five countries: Belgium and Italy in Europe, China in Asia, and Mexico and the USA in North America. Their results showed that food neophobia and disgust were negatively associated with the will to eat insects, regardless of them being processed or whole.

EIs also play a part in society for economic reasons and ensure the livelihood of rural communities, as well as cultural or religious traditions [5,6,7]. On the other hand, in Western countries, the consumption of EIs is a more recent phenomenon, motivated by their contribution to sustainability, nutritional value and benefits [8]. In addition, their gastronomic value has been disseminated through renowned chefs, thus contributing to their valorisation as a gourmet food providing palatable new experiences [9,10].

Apart from their previously described roles, EIs contribute to the environment by providing biodiversity [11] and by being sustainable sources of animal protein. They require fewer land areas, they produce lower amounts of greenhouse gases, and they consume lower quantities of water and feed [12]. For these reasons, the FAO of the United Nations recommends their consumption as a part of the global strategy for sustainability and food safety [13].

EIs have a rich composition in several macro- and micronutrients: for example, proteins and a diversity of amino acids, including essential amino acids, fat and, particularly, unsaturated fatty acids; carbohydrates, including dietary fibre; and vitamins, such as riboflavin, pantothenic acid, biotin, and folic acid, and minerals such as copper, iron, magnesium, manganese, phosphorus, selenium, and zinc [14]. Additionally, EIs contain bioactive compounds with benefits for human health, as a number of recent studies have demonstrated [15,16]. Among the bioactive compounds are the bioactive peptides from insects [17], which carry benefits for human health due to their antioxidant, antimicrobial and antidiabetic properties. Also, these peptides exhibit the capacity to inhibit the angiotensin I-converting enzyme. Moreover, they can be advantageous to the food systems, and they can be used as food ingredients to produce functional foods [17].

The nutritional and bioactive properties of EIs confirm their long-time use for healing purposes among entomophagic communities. The therapeutic potentialities of EIs have been preserved by traditional healers, but in the present day, the therapeutic knowledge regarding EIs can assist modern medicine [18,19,20,21,22]. A work by Devi et al. [18] focused on the role of EIs on traditional medicine as well as their value for achieving modern human health. In Northeast India, some people possess a traditional knowledge about the value of insects as medicine for healing a number of diseases. Around 90 species of EIs are reported as being used in medicine by a number of indigenous communities [18]. Concerning the modern utilisation of EIs for healing purposes, a work by Nowakowski et al. [23] highlighted the potential benefits for human health associated with EIs. In the work, the roles of EIs in the management of chronic diseases like diabetes, cancer and cardiovascular disease were pointed out, as well as their roles in enhancing immune function [23]. It was reported that an increase in probiotic bacterium *Bifidobacterium animalis* and a reduction in plasma tumour necrosis factor was as a result of consuming cricket powder. As a result, an associated effect has been described to improve health in various diseases, like rheumatoid arthritis, inflammatory bowel diseases, multiple sclerosis, and multiple types of cancer [24,25].

Nevertheless, the use of EIs must be risk-free, and therefore, some attention must also be paid to the possible and nutritional or harmful effects of EI, such as the risk they can pose to health. Among the anti-nutritional factors, the presence of compounds like oxalates, phytates, or saponins, which diminish the absorption of nutrients like proteins or minerals, can be highlighted in some EIs. Other hazards may result from microbial or pesticide contamination [15,26,27]. Mwelwa et al. [28] investigated the bio-transfer of heavy metals along the EI food chain, from soil to plant to EIs to humans at the end of the chain. They concluded that there is a potential bio-transfer of heavy metals along the EIs chain when insects are harvested in environments polluted with heavy metals, thus posing a serious risk to human health. However, in Western countries, where the EIs are not harvested in the wild but reared in farms, the production is controlled, and EIs are safe for human consumption [29].

Considering the sustainability implications of consuming EIs, as well as their nutritional and health effects, knowledge plays a fundamental role in their acceptance and wider consumption. The consumption of EIs may be intensely shaped by factors of cultural nature, personal traits, expectations, and knowledge about their effects on nutrition and health and their environmental and economic impacts. Nevertheless, although it is very common to find studies about the motivations for the consumption of EIs in the scientific literature [30,31,32,33], studies focusing on knowledge about EIs are much more scarce, and they can assume importance in shaping the consumer’s will to accept EIs as part of their usual diets. Therefore, the aim of this study was to investigate how the participants from 14 different countries perceive some nutritional facts and health effects of EIs, and how the level of information can be influenced by sociodemographic characteristics such as country, age, sex, level of education or living environment.

## 2. Materials and Methods

### 2.1. Instrument and Data Collection

This work was conducted through a questionnaire survey in the ambit of the international project EISuFood whose instrument was validated in a previous work [33]. Ethical approval was obtained from the Ethics Committee of the Polytechnic University of Viseu (Ref. Nº 45/SUB/2021).

The questionnaire contained 10 questions about nutritional and anti-nutritional aspects of EIs and 10 questions about the positive or negative health effects of EIs (Table 1). The respondents had to answer the 20 questions using a 5-point central Likert scale: 1 = strongly disagree, 2 = disagree, 3 = neither agree nor disagree, 4 = agree, and 5 = strongly agree [34].

Data collection took place simultaneously in 14 countries: Brazil, Croatia, Greece, Latvia, Lebanon, Lithuania, Mexico, Poland, Portugal, Romania, Serbia, Slovenia, Spain and Turkey. All ethical principles were followed for the data collection, including informed consent, guarantee of anonymity of the responses and the right to cancel participation during the filling of the questionnaire. Data collection took place using online tools, only involving adult citizens (aged 18 years old or over) who expressed their informed consent and the will to participate voluntarily and without any monetary reward.

### 2.2. Sample Characterisation

**Figure 1:**
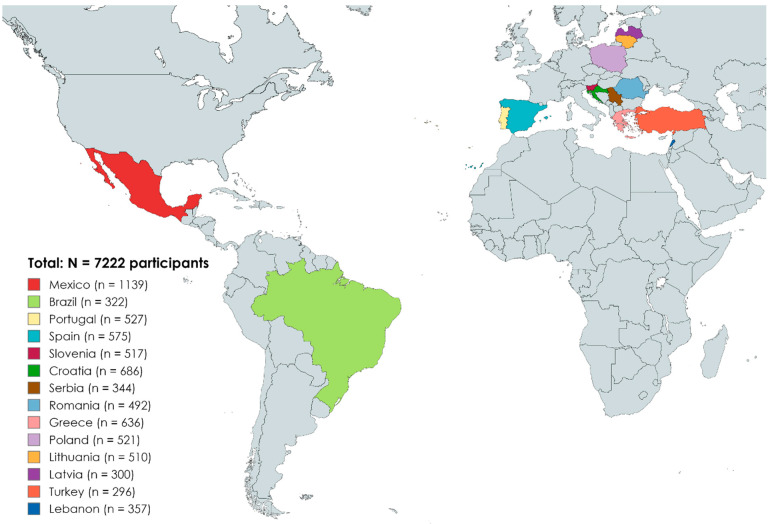
Geographical distribution of the participants in the study (N = 7222).

A total of 7222 validated answered questionnaires were obtained, with a geographical distribution as indicated in Figure 1. This study included participants from the American continent (Mexico in North America and Brazil in South America), Western Europe (Portugal and Spain in the Iberian Peninsula), Northern Europe (Poland, Latvia and Lithuania), Central Europe (Romania, Serbia, Croatia and Slovenia), South Europe (Greece) and the Middle East (Turkey and Lebanon).

The participants were mostly female (63.5%), with a lower participation of males (35.9%). The distribution of the participants according to age classes was 47.4% aged between 18 and 30 years, 37.0% aged between 31 and 50 years, and 15.6% aged 51 years or more. The majority lived in urban areas (65.6%), but some lived in rural environments (19.1%) or suburban areas (15.3%). With respect to education level, 35.8% did not achieve a university level, 32.3% completed a university degree, and 31.9% had completed post-graduate studies (Masters or Doctorate degrees).

### 2.3. Statistical Analysis

For all statistical analyses, we used SPSS Version 28 (IBM, Inc., Armonk, NY, USA). Some basic descriptive statistics were used, such as frequency tables for sociodemographic variables and for the 20 questions used in the questionnaire to assess the level of knowledge about EIs’ nutritional value and health effects.

An exploratory analysis of the items used in the questionnaire was performed using factor analysis (FA) with the method of principal components (PCs). Prior to the analysis, it was confirmed whether the data were suitable for the FA analysis [35]. One of the criteria related to the correlation matrix should identify possible correlations between the variables. The second criterion was the value of the Kaiser–Meyer–Olkin (KMO) measure of adequacy, whose values should indicate adequacy according to the following scale: excellent for 0.9 ≤ KMO ≤ 1.0, good for 0.8 ≤ KMO < 0.9, acceptable for 0.7 ≤ KMO < 0.8, tolerable for 0.6 ≤ KMO < 0.7, bad for 0.5 ≤ KMO < 0.6, and unacceptable for KMO < 0.5. The third criterion was the significance of Bartlett’s test, considering a 5% level of significance [36].

After the confirmation that the data were suitable to apply the FA, we performed the analysis with the PC method and Varimax rotation. The Kaiser normalization was used to extract the relevant factors with eigenvalues above 1. To investigate the percentage of variance explained by the factors extracted, the communalities were used [35]. When classifying the variables (questions) according to the factors extracted by the FA, the variables with factor loadings whose absolute value was lower than 0.4 were excluded for having a low relevance to the factor [37,38]. To quantify the internal consistency of each factor, the standard measure of Cronbach’s alpha (α) was used [35,39], whose values are interpreted as follows: desirable values should be over 0.7 and preferably over 0.8, which corresponds to a very good internal consistency. Nevertheless, some authors also state that values over 0.5 could be acceptable [40,41,42].

The data collected were subjected to a one-way analysis of variance (ANOVA) to evaluate possible differences across sociodemographic groups, and in order to identify which means were significantly different from the others, the Tukey HSD (honestly significant difference) post-hoc test was used. A 5% level of significance was used.

## 3. Results

### 3.1. Level of Agreement of the Participants with the Questions

Table 2 shows the percentage of responses obtained for each of the questions according to the five scores used to measure the level of agreement (ranging from 1 = strongly disagree to 5 = strongly agree).

The results indicated that for most of the questions, many participants manifested a neutral opinion by replying neither agree nor disagree (percentages of score 3 ranging from a minimum of 25.4% for question Q3 to a maximum of 66.0% for Q10). For most of the items, the participants scored 4 or 5, corresponding to agreement or strong agreement, respectively, while for a few questions, the participants expressed disagreement (higher percentages of scores 1 and 2), like for questions Q1, Q4, Q13 or Q15.

### 3.2. Factor Analysis

The first phase was to verify the assumptions that the data were suitable for applying FA. The three criteria were:

1. The correlation matrix must identify possible correlations between the variables;
2. The value of the Kaiser–Meyer–Olkin (KMO) measure of adequacy must be as close as possible to one;
3. The Bartlett’s test must be significant (*p* < 0.05).

The first one was confirmed as the correlation matrix showed some correlations between the variables (seven values of r higher than 0.5, with the highest being 0.642 for the correlation between Q6 and Q8). Regarding the second criterion, the value of KMO was found to be 0.906, which corresponds to excellent [36]. Finally, the third criterion was also confirmed as Bartlett’s test was significant (*p* < 0.001), confirming the rejection of the null hypothesis “H0: The correlation matrix is equal to the identity matrix”.

After confirmation of the assumptions, FA was applied as described earlier. The anti-image matrix revealed that all values of the correlations were over 0.5. Therefore, neither of the variables should be excluded from the analysis (the lowest of the values was 0.808 for variable Q15, and the highest was 0.939 for variable Q5).

The commonalities, which indicate the percentage of variance of each item explained by the solution, were calculated for each of the 20 variables (Table 3). The results in Table 3 reveal that practically all variables had a minimum of 50% of their variance explained by the solution, with variable Q10 having the lowest value (43.8%), while variable Q8 had the highest percentage of variance explained (68.1%).

Table 4 presents the results obtained for the FA, whose solution converged in seven iterations and contains four factors, which in total explain 55.2% of variance: factor F1 (eigenvalue = 5.661, %VE = 19.3%), factor F2 (eigenvalue = 2.940, %VE = 13.6%), factor F3 (eigenvalue = 1.396, %VE = 12.2%) and factor F4 (eigenvalue = 1.051, %VE = 10.1%). This solution included all 20 items as they all had loadings higher than 0.4 (absolute value) in at least one factor. The grouping structure resulting from the FA can be interpreted as follows: factor F4 accounting for items related to contamination and harmful components (CHCs), factor F2 grouping the items associated with negative perception and risks (NPRs), factor F3 corresponding to the items related to safety and benefits of insects (SBIs) and factor F1 accounting for the items that address the nutritive value (NV).

All factor loadings were above 0.5, varying in the ranges of 0.518–0.763 for F1; 0.600–0.729 for F2; 0.552–0.696 for F3; and 0.538–0.684 for F4. High loading factors indicate that the items strongly contribute to the factors’ definition. The item with the lowest loading was Q3, which was related to the high protein content of EIs (loading of 0.518 to factor F1). The item with the highest loading was Q8, concerning insects containing minerals of dietary relevance (loading of 0.763 to factor F1), meaning that this question is the most strongly associated with the respective factor.

The measure considered to evaluate the internal consistency of the items in each factor was Cronbach’s alpha (α) [35], which assumed values considered acceptable for factors F2—NPRs and F4—CHCs (α = 0.688 and α = 0.626, respectively) while being good for factor F3—SBIs (α = 0.724) and very good for factor F1—NV (α = 0.851 considering all the eight variables, or an even higher α = 0.862 if item Q10 is removed) [40,41,42].

Based on these results of the internal consistency analysis, the final FA solution was considered with only 19 of the items, i.e., excluding item Q10 from factor F1.

### 3.3. Sociodemographic Variations

Based on the results of the FA, the average values of the scores for the variables in the four factors were calculated for each participant, and those values were then compared between groups according to the sociodemographic variables considered: country, age, sex, education level, and living environment (Table 5). The results showed significant country differences and significant differences across the age groups (*p* < 0.05 in all cases) for all four factors. Regarding sex or education groups, significant differences were observed for factors F1 (NV), F2 (NPRs) and F3 (SBIs) but not for F4 (CHCs). Finally, for living environment groups, significant differences were only found for factors F1 (NV) and F2 (NPRs).

## 4. Discussion

The results of this research revealed a high difficulty among the participants to express a negative or positive opinion about nutritional and health facts related to the consumption of EIs, confirming that in this list of 14 countries, the general population is still very little informed about EIs. It is a fact that most of the countries at study are European, and therefore, they belong to the list of countries where entomophagy is not a part of the diet tradition. Additionally, in Western countries, there is still some reluctance to consume EIs, partly due to people being unaccustomed to it and partly owing to neophobia and disgust manifested by many European citizens [32,43,44]. In contrast, other countries included in the study have some traditions of entomophagy, such as Mexico. However, not all Mexican populational groups are entomophagous, and there are areas where consumers also do not have a tradition of eating insects. The work by Escalante-Aburto et al. [45] refers that Mexico is a multi-diverse country with some tradition of entomophagy, but differences were encountered within different regions of Mexico; in the south and centre, people consume more EIs, while in the north, the practice is not so usual. A study by Gómez-Corona et al. [46] revealed that some Mexican citizens are insect eaters and others are not, but the representation that they have about EIs is similar nonetheless. Additionally, the same study highlighted that sustainability, convenience and affordability were the principal motivations for consuming insects.

The participants who were able to manifest an agreement/disagreement with the statements of the questionnaire revealed to have some knowledge about most of the topics addressed.

Most participants who expressed an opinion were aware of the nutritional value of EIs and the presence of a diversity of macro- and micronutrients important for the human body. Specifically, the participants agreed that Q1. Insects are a good source of energy [47]; Q2. Insects have high protein content [48]; Q5. Insects provide essential amino acids necessary for humans [48]; Q6. Insects contain group B vitamins [47]; Q7. Insects contain dietary fibre [48]; Q8. Insects contain minerals of nutritional interest, such as calcium, iron and magnesium [47]; and Q9. Insects contain fat, including unsaturated fatty acids [47,49]. All these food components are present in EIs in variable proportions, depending on the species and other growing variables [50,51,52].

With respect to the presence of anti-nutrients, the participants showed some knowledge about this possibility by agreeing with question Q10. Insects contain anti-nutrients, such as oxalates and phytic acid [53]. The presence of some components, like oxalates, phytates or saponins, that eventually compromise the absorption of other beneficial nutrients such as proteins or minerals, constitutes a drawback in some EIs, but this is not general for all and depends on the species [15].

Concerning the health effects of EIs, the participants were also in agreement with most of the questions. Q12. Insects are used by some people in traditional medicine [47]; Q17. In certain countries, insects are approved officially for therapeutic treatment [49]; and Q18. Insects contain bioactive compounds that are beneficial to human health [49]. All of these questions present facts that are known by some of the participants in the study. The use of insects in traditional medicine in some areas of the globe is a long-time practice, as discussed by Zhang et al. [54], Siddiqui et al. [55], and Figueiredo et al. [56]. The ethnoentomological tradition is well documented across Africa, Asia (India, China and South Korea), Latin America and Mexico. The insects are used as food (entomophagy) and medicine (entomotherapy) and are used in rite-of-passage rituals. Some of the beneficial health effects described include improving immune function and treating rheumatism, anaemia, cancer and skin diseases [54]. Many of these traditional usages of insects have been studied and confirmed in light of present science. In a recent review, Tanga and Ekesi [57] highlight some therapeutic usages of EIs, which include the enhancement of immune functions and protection of the gastrointestinal system, as well as anticancer, antioxidant and anti-inflammatory effects; antibacterial activity; regulation of serum lipids and glucose, lowering of blood pressure, and reduced risk of developing cardiovascular diseases.

A high fraction of participants expressed disagreement with some questions, such as Q1, Q4, Q13 and Q15. With respect to Q1. Insects have poor nutritional value [47], this statement is in fact not true, and many studies have highlighted the high nutritional value of EIs, for example, in the reviews conducted by Nowak et al. [50] or Baionao [29]. Question Q4. Insect proteins are of poorer quality compared with other animal species [47] is also a false statement as EIs contain high amounts of protein and a diversity of amino acids, including essential amino acids of good nutritional value, although they vary depending on the insect [16,58,59]. Questions Q13. Eating insects poses a substantial risk to human health [60] and Q15. Insects and insect-based foods are often infected by pathogens and parasites [61] also obtained scores for disagreement from a high fraction of the participants, revealing that they considered EIs to be safe for consumption on a general basis. When EIs are part of controlled food chains, like in the Western countries markets, they are safe for human consumption, and they do not pose risks to human health. On the other hand, EIs can pose some risks only if they are not produced, handled, and processed with respect to food safety issues or collected from the wild and manipulated in a non-hygienic manner [62,63]. For this matter, it was observed that the participants were relatively well informed about safety and regulations regarding EIs, as expressed in question Q11. There are appropriate regulations to guarantee the food safety of edible insects [47], i.e., Q14. Industrial-processed insect products are hygienic and safe [60]. Nevertheless, these regulations, as well as food chain security measures, are variable among regions, with the European Union being particularly strict in comparison with other global markets.

Other subjects that the participants revealed to have some knowledge about were Q19. Insects are potential sources of allergens [63]; Q16. Insects collected from the wild may be contaminated with pesticide residues [63]; and Q20. Aflatoxins, which are carcinogens, can be present in insects [63]. These aspects can in fact compromise the health of insect eaters, but their occurrence is generally low and variable according to many factors, ranging from the species to the sanitary measures used for their production and transformation.

In addition, we found four underlying dimensions that explain the relationships between the items about the nutrition and health of the EIs, one containing the questions about the nutritive value (NV) of EIs, a second dimension joining the questions about the negative perception and risks (NPRs) associated with EI, a third gathering the questions linked with the safety and benefits of insects (SBIs), and finally the last containing the questions about the contamination and harmful components (CHCs) possibly present in EIs. It was observed that knowledge on these four subjects was related to the sociodemographic characteristics of the participants. While living environment influenced knowledge in two of the four groups, sex and education influenced knowledge in three groups; age and country were the sociodemographic variables with the greatest influence, with differences in knowledge in all four groups. The country would be expected to be a major determinant for knowledge about EIs, given the diversity of social and cultural influences, particularly on a subject that is so differently perceived among insect-eating countries and Western countries. The review by Florença et al. [8] showed some important differences between the motivations for consumption of EIs by citizens from these two types of countries. It is, therefore, natural to assume that among societies where insects are regularly consumed, there is a wider dissemination of information about EIs. This work confirmed a higher average level of knowledge for Mexican and Lebanese participants, as well as differences among European countries, with Spanish and Polish participants being better informed about the nutritive value and health effects of EIs. Previous work has shown that knowledge is a valuable instrument for the acceptance of insects as food in communities where there is no tradition of eating insects [64].

## 5. Conclusions

This work revealed that although a high percentage of participants had a neutral opinion (neither agreed nor disagreed) about the facts concerning the nutritive value and health effects of EIs, many participants still expressed strong agreement or disagreement about the nutritional value and health effects of EIs, which may indicate that they are well informed. Also, this work revealed four groups of knowledge: one related to the nutritional composition of EIs, another related to a negative perception of the participants about EIs and possible risks they associate with them, a third about the safety and benefits of consuming EIs, and a fourth group about the possible presence of contaminants and harmful components in EIs. Finally, it was concluded that all sociodemographic variables studies have some level of relation with the knowledge about the nutritional and health effects of EIs, with variables such as age and country being the most influential. As such, knowledge is assumed to be a pivotal ally to incentivise the consumption of EI, with benefits not only for the environment but, most importantly, for nutritional purposes and the beneficial health effects that EIs may provide. At any rate, it is not negligible that care must also be given to fully understand the possible negative effects of EIs, such as the presence of allergens or other harmful substances.

The promotion of consumption of EI as a sustainable alternative assumes a pivotal role in food security, particularly for socially vulnerable individuals, not only in developing countries but also in other areas, and even for people residing in European countries (like the refugees, for example); homeless people; and those living on the outskirts, in rural areas or those experiencing drought and calamities, among others, all over the globe. Finally, longitudinal research could provide deeper insights into the dynamics of knowledge acquisition and how it affects the acceptance of insects as a sustainable and healthy food source. Furthermore, while the study found substantial differences depending on sociodemographic characteristics such as age and country of residence/origin, other important variables, such as cultural attitudes toward entomophagy or exposure to insect-based meals, were not thoroughly investigated. Therefore, future research could look at these characteristics to have a better understanding of the causes and barriers to insects being accepted as a viable food source in a variety of populations.

## Figures and Tables

**Table 1:** Questions used to assess knowledge about nutrition and the health effects of EI.

N°	Item Description
1	Insects have poor nutritional value
2	Insects are a good source of energy
3	Insects have high protein content
4	Insect proteins are of poorer quality compared with other animal species
5	Insects provide essential amino acids necessary for humans
6	Insects contain group B vitamins
7	Insects contain dietary fibre
8	Insects contain minerals of nutritional interest, such as calcium, iron and magnesium
9	Insects contain fat, including unsaturated fatty acids
10	Insects contain anti-nutrients, such as oxalates and phytic acid
11	There are appropriate regulations to guarantee food safety of edible insects
12	Insects are used by some people in traditional medicine
13	Eating insects poses a substantial risk to human health
14	Industrial-processed insect products are hygienic and safe
15	Insects and insect-based foods are often infected by pathogens and parasites
16	Insects collected from the wild may be contaminated with pesticide residues
17	In certain countries, insects are approved officially for therapeutic treatment
18	Insects contain bioactive compounds beneficial to human health
19	Insects are potential sources of allergens
20	Aflatoxins, which are carcinogens, can be present in insects

**Table 2:** Frequency of responses to the 20 questions about the nutrition and health of EIs.

N°	Item Description	% of Responses in Each Score ^1^				
		1	2	3	4	5
1	Insects have poor nutritional value	26.8	32.6	30.2	7.1	3.3
2	Insects are a good source of energy	4.5	9.2	33.0	35.1	18.2
3	Insects have high protein content	2.9	4.0	25.4	39.8	27.9
4	Insect proteins are of poorer quality compared with other animal species	17.0	24.5	43.8	10.7	4.0
5	Insects provide essential amino acids necessary for humans	3.4	6.1	52.3	25.7	12.5
6	Insects contain group B vitamins	3.3	6.3	64.1	18.3	8.0
7	Insects contain dietary fibre	5.3	80.0	51.9	23.7	11.1
8	Insects contain minerals of nutritional interest, such as calcium, iron and magnesium	3.3	5.4	51.9	28.3	11.1
9	Insects contain fat, including unsaturated fatty acids	5.3	12.0	56.8	18.0	7.9
10	Insects contain anti-nutrients, such as oxalates and phytic acid	6.3	10.1	66.0	13.1	4.5
11	There are appropriate regulations to guarantee food safety of edible insects	7.7	14.0	44.4	24.2	9.7
12	Insects are used by some people in traditional medicine	3.1	5.4	31.5	42.2	17.8
13	Eating insects poses a substantial risk to human health	15.7	30.4	36.3	12.9	4.7
14	Industrial-processed insect products are hygienic and safe	4.5	9.0	40.9	32.3	13.3
15	Insects and insect-based foods are often infected by pathogens and parasites	11.5	21.0	48.5	13.7	5.3
16	Insects collected from the wild may be contaminated with pesticide residues	3.1	7.2	33.6	39.4	16.7
17	In certain countries, insects are approved officially for therapeutic treatment	2.5	5.5	54.1	28.4	9.5
18	Insects contain bioactive compounds beneficial to human health	3.2	5.7	53.2	27.7	10.2
19	Insects are potential sources of allergens	4.5	10.3	54.3	22.6	8.3
20	Aflatoxins, which are carcinogens, can be present in insects	5.7	12.3	63.4	13.9	4.7

^1^ Scale of the scores: 1 = strongly disagree, 2 = disagree, 3 = neither agree nor disagree, 4 = agree, and 5 = strongly agree.

**Table 3:** Communalities for 20 questions about nutrition and health of EI.

Question	%VE ^1^	Question	%VE ^1^	Question	%VE ^1^	Question	%VE ^1^
Q1	54.2	Q6	60.5	Q11	56.1	Q16	55.1
Q2	52.4	Q7	54.0	Q12	52.1	Q17	54.5
Q3	63.5	Q8	68.1	Q13	53.8	Q18	55.5
Q4	55.8	Q9	55.7	Q14	47.8	Q19	53.1
Q5	62.6	Q10	43.8	Q15	55.3	Q20	50.8

^1^ %VE = Percentage variance explained.

**Table 4:** The solution obtained by the factor analysis.

Factor	%VE ^1^	Questions	Loadings	Factor Name	Cronbach’s Alpha (α)
F1	19.3	Q2. Insects are a good source of energy	0.551	Nutritive value (NV)	0.851
		Q3. Insects have high protein content	0.518		0.862 ^2^
		Q5. Insects provide essential amino acids necessary for humans	0.683		
		Q6. Insects contain group B vitamins	0.737		
		Q7. Insects contain dietary fibre	0.718		
		Q8. Insects contain minerals of nutritional interest (Ca, Fe, Mg)	0.763		
		Q9. Insects contain fat, including unsaturated fatty acids	0.722		
		[Q10. Insects contain anti-nutrients (oxalates and phytic acid)]	[0.526]		
F2	13.6	Q1. Insects have poor nutritional value	0.718	Negative perception and risks (NPRs)	0.688
		Q4. Insect proteins are of poorer quality than other animals	0.729		
		Q13. Eating insects poses a substantial risk to human health	0.600		
F3	12.2	Q11. There are regulations to guarantee food safety of insects	0.696	Safety and benefits of insects (SBIs)	0.724
		Q12. Insects are used by some people in traditional medicine	0.673		
		Q14. Industrial-processed insect products are hygienic and safe	0.557		
		Q17. In certain countries, insects are approved for therapeutics	0.687		
		Q18. Insects contain beneficial bioactive compounds	0.552		
F4	10.1	Q15. Insects/insect -foods can be infected by pathogens/parasites	0.538	Contamination and harmful components (CHCs)	0.626
		Q16. Insects from the wild may be contaminated with pesticides	0.679		
		Q19. Insects are potential sources of allergens	0.684		
		Q20. Aflatoxins, which are carcinogens, can be present in insects	0.674		

^1^ VE = Variance explained. ^2^ The value of Cronbach’s alpha increases if item Q10 is removed.

**Table 5:** Comparison of the mean values for the four factors (F1 to F4) across sociodemographic groups.

Variables	Groups	Mean ± SD Factor F1 (NV) ^1^	Mean ± SD Factor F2 (NPRs) ^1^	Mean ± SD Factor F3 (SBIs) ^1^	Mean ± SD Factor F4 (CHCs) ^1^
Country	Brazil	3.31 ± 0.63 ^bcd^	2.57 ± 0.77 ^de^	3.34 ± 0.63 ^bcd^	3.20 ± 0.57 ^bcde^
	Croatia	3.09 ± 0.77 ^ab^	2.84 ± 0.79 ^hi^	3.31 ± 0.73 ^abc^	3.31 ± 0.66 ^e^
	Greece	3.21 ± 0.63 ^a^	2.68 ± 0.70 ^efgh^	3.30 ± 0.62 ^ab^	3.28 ± 0.63 ^e^
	Latvia	3.30 ± 0.45 ^bc^	2.63 ± 0.63 ^efg^	3.35 ± 0.46 ^bcd^	3.25 ± 0.43 ^de^
	Lebanon	3.76 ± 0.57 ^f^	2.80 ± 0.76 ^ghi^	3.85 ± 0.66 ^f^	3.56 ± 0.61 ^f^
	Lithuania	3.45 ± 0.62 ^de^	2.61 ± 0.69 ^ef^	3.31 ± 0.46 ^abc^	3.13 ± 0.60 ^bcd^
	Mexico	3.70 ± 0.72 ^f^	2.04 ± 0.87 ^a^	3.45 ± 0.63 ^de^	2.96 ± 0.66 ^a^
	Poland	3.51 ± 0.55 ^e^	2.28 ± 0.69 ^bc^	3.49 ± 0.51 ^e^	3.18 ± 0.52 ^bcde^
	Portugal	3.30 ± 0.65 ^bc^	2.54 ± 0.69 ^de^	3.29 ± 0.64 ^ab^	3.11 ± 0.56 ^bcd^
	Romania	3.26 ± 0.60 ^bc^	2.61 ± 0.70 ^ef^	3.33 ± 0.63 ^abcd^	3.11 ± 0.63 ^bc^
	Serbia	3.21 ± 0.67 ^ab^	2.76 ± 0.70 ^fghi^	3.23 ± 0.73 ^ab^	3.07 ± 0.70 ^ab^
	Slovenia	3.39 ± 0.64 ^cde^	2.43 ± 0.80 ^cd^	3.44 ± 0.67 ^cde^	3.08 ± 0.59 ^ab^
	Spain	3.47 ± 0.69 ^e^	2.12 ± 0.78 ^ab^	3.47 ± 0.66 ^de^	2.96 ± 0.62 ^a^
	Turkey	3.21 ± 0.66 ^ab^	2.85 ± 0.66 ^i^	3.20 ± 0.59 ^a^	3.24 ± 0.63 ^cde^
	*p*-value ^2^	<0.001	<0.001	<0.001	<0.001
Age group	18–30 y	3.42 ± 0.69 ^b^	2.52 ± 0.81 ^b^	3.40 ± 0.65 ^b^	3.16 ± 0.64 ^b^
	31–50 y	3.40 ± 0.68 ^b^	2.44 ± 0.79 ^a^	3.40 ± 0.63 ^b^	3.11 ± 0.61 ^a^
	51+ y	3.30 ± 0.67 ^a^	2.56 ± 0.77 ^b^	3.34 ± 0.64 ^a^	3.19 ± 0.63 ^b^
	*p*-value ^2^	<0.001	<0.001	0.018	<0.001
Sex	Female	3.36 ± 0.66 ^a^	2.53 ± 0.76 ^a^	3.38 ± 0.63 ^a^	3.15 ± 0.62 ^a^
	Male	3.46 ± 0.72 ^b^	2.44 ± 0.86 ^ab^	3.40 ± 0.66 ^a^	3.14 ± 0.65 ^a^
	No answer	3.38 ± 0.78 ^a^	2.25 ± 0.78 ^b^	3.58 ± 0.77 ^b^	3.04 ± 0.61 ^a^
	*p*-value ^2^	<0.001	<0.001	0.042	0.328
Living environment	Rural	3.33 ± 0.68 ^a^	2.57 ± 0.80 ^b^	3.38 ± 0.69 ^a^	3.17 ± 0.66 ^a^
	Urban	3.40 ± 0.69 ^b^	2.46 ± 0.80 ^a^	3.39 ± 0.63 ^a^	3.14 ± 0.62 ^a^
	Suburban	3.44 ± 0.67 ^b^	2.53 ± 0.81 ^b^	3.39 ± 0.64 ^a^	3.17 ± 0.64 ^a^
	*p*-value ^2^	<0.001	<0.001	0.757	0.071
Education level	Post-graduate	3.44 ± 0.63 ^b^	2.44 ± 0.77 ^a^	3.41 ± 0.59 ^b^	3.16 ± 0.57 ^a^
	University degree	3.43 ± 0.70 ^b^	2.47 ± 0.80 ^a^	3.43 ± 0.66 ^b^	3.14 ± 0.66 ^a^
	No university degree	3.32 ± 0.71 ^a^	2.56 ± 0.82 ^b^	3.32 ± 0.66 ^a^	3.14 ± 0.66 ^a^
	*p*-value ^2^	<0.001	<0.001	<0.001	0.489

^1^ Mean values in the same factor with different superscript letters are significantly different across sociodemographic groups. ^2^ Significance of the ANOVA test with Tukey’s post-hoc test (*p* < 0.05).

## Data Availability

Data are available from the corresponding author upon request.

## References

[1] Van Huis A., Halloran A., Itterbeeck J.V., Klunder H., Vantomme P. (2022). How Many People on Our Planet Eat Insects: 2 Billion?. J. Insects Food Feed.

[2] Payne C., Caparros Megido R., Dobermann D., Frédéric F., Shockley M., Sogari G., Sogari G., Mora C., Menozzi D. (2019). Insects as Food in the Global North—The Evolution of the Entomophagy Movement. Edible Insects in the Food Sector: Methods, Current Applications and Perspectives.

[3] Mancini S., Sogari G., Espinosa Diaz S., Menozzi D., Paci G., Moruzzo R. (2022). Exploring the Future of Edible Insects in Europe. Foods.

[4] Sogari G., Riccioli F., Moruzzo R., Menozzi D., Tzompa Sosa D.A., Li J., Liu A., Mancini S. (2023). Engaging in Entomophagy: The Role of Food Neophobia and Disgust between Insect and Non-Insect Eaters. Food Qual. Prefer..

[5] Aigbedion-Atalor P.O., Fening K.O., Adeyemi A.O., Idemudia I., Ojukwu K.C., Nwobodo M.A., Sunday O., Isiogu N.C., Oke A.O. (2024). Regenerative Edible Insects for Food, Feed, and Sustainable Livelihoods in Nigeria: Consumption, Potential and Prospects. Future Foods.

[6] Bisconsin-Júnior A., Rodrigues H., Behrens J.H., da Silva M.A.A.P., Mariutti L.R.B. (2022). “Food Made with Edible Insects”: Exploring the Social Representation of Entomophagy Where It Is Unfamiliar. Appetite.

[7] Lange K.W., Nakamura Y. (2021). Edible Insects as Future Food: Chances and Challenges. J. Future Foods.

[8] Florença S.G., Guiné R.P.F., Gonçalves F.J.A., Barroca M.J., Ferreira M., Costa C.A., Correia P.M.R., Cardoso A.P., Campos S., Anjos O. (2022). The Motivations for Consumption of Edible Insects: A Systematic Review. Foods.

[9] Van Huis A., Rumpold B. (2023). Strategies to Convince Consumers to Eat Insects? A Review. Food Qual. Prefer..

[10] Halloran A., Flore R., Mercier C. (2015). Notes from the ‘Insects in a Gastronomic Context’ Workshop in Bangkok, Thailand. J. Insects Food Feed.

[11] Evangelista D.A., Scheiner S.M. (2024). Insects, 60% of All Biodiversity. Encyclopedia of Biodiversity.

[12] Guiné R.P.F., Correia P., Coelho C., Costa C.A. (2021). The Role of Edible Insects to Mitigate Challenges for Sustainability. Open Agric..

[13] FAO (2021). Looking at Edible Insects from a Food Safety Perspective-Challenges and Opportunities for the Sector.

[14] Sánchez-Estrada M.d.l.L., Aguirre-Becerra H., Feregrino-Pérez A.A. (2024). Bioactive Compounds and Biological Activity in Edible Insects: A Review. Heliyon.

[15] Rivas-Navia D.M., Dueñas-Rivadeneira A.A., Dueñas-Rivadeneira J.P., Aransiola S.A., Maddela N.R., Prasad R. (2023). Bioactive Compounds of Insects for Food Use: Potentialities and Risks. J. Agric. Food Res..

[16] da Silva Lucas A.J., de Oliveira L.M., da Rocha M., Prentice C. (2020). Edible Insects: An Alternative of Nutritional, Functional and Bioactive Compounds. Food Chem..

[17] Zielińska E., Baraniak B., Karaś M., Rybczyńska K., Jakubczyk A. (2015). Selected Species of Edible Insects as a Source of Nutrient Composition. Food Res. Int..

[18] Devi W.D., Bonysana R., Kapesa K., Mukherjee P.K., Rajashekar Y. (2023). Edible Insects: As Traditional Medicine for Human Wellness. Future Foods.

[19] Verheyen G.R., Pieters L., Maregesi S., Van Miert S. (2021). Insects as Diet and Therapy: Perspectives on Their Use for Combating Diabetes Mellitus in Tanzania. Pharmaceuticals.

[20] Ratcliffe N.A., Mello C.B., Garcia E.S., Butt T.M., Azambuja P. (2011). Insect Natural Products and Processes: New Treatments for Human Disease. Insect Biochem. Mol. Biol..

[21] Tan J., Tian Y., Cai R., Yi T., Jin D., Guo J. (2019). Antiproliferative and Proapoptotic Effects of a Protein Component Purified from Aspongopus Chinensis Dallas on Cancer Cells In Vitro and In Vivo. Evid. Based Complement. Altern. Med..

[22] Rethinavelu G., Manoharan L., Krishnamoorthy S., Baskaran N., Sivanandham V. (2023). Edible Lichens and Its Unique Bioactives: A Review of Its Pharmacological and Food Applications. Food Humanit..

[23] Nowakowski A.C., Miller A.C., Miller M.E., Xiao H., Wu X. (2022). Potential Health Benefits of Edible Insects. Crit. Rev. Food Sci. Nutr..

[24] Chu W.-M. (2013). Tumor Necrosis Factor. Cancer Lett..

[25] Stull V.J., Finer E., Bergmans R.S., Febvre H.P., Longhurst C., Manter D.K., Patz J.A., Weir T.L. (2018). Impact of Edible Cricket Consumption on Gut Microbiota in Healthy Adults, a Double-Blind, Randomized Crossover Trial. Sci. Rep..

[26] Belluco S., Losasso C., Maggioletti M., Alonzi C.C., Paoletti M.G., Ricci A. (2013). Comprehensive Reviews in Food Science and Food Safety.

[27] Schönleben A.M., Yin S., Strak E., Johnson A., Belova L., Ait Bamai Y., van Nuijs A.L.N., Poma G., Covaci A. (2024). Stable Isotope Ratios and Current-Use Pesticide Levels in Edible Insects: Implications on Chemical Food Safety. Food Res. Int..

[28] Mwelwa S., Chungu D., Tailoka F., Beesigamukama D., Tanga C.M. (2023). Data to Understand the Biotransfer of Heavy Metals along the Soil-Plant-Edible Insect-Human Food Chain in Africa. Data Brief.

[29] Baiano A. (2020). Edible Insects: An Overview on Nutritional Characteristics, Safety, Farming, Production Technologies, Regulatory Framework, and Socio-Economic and Ethical Implications. Trends Food Sci. Technol..

[30] Hopkins I., Farahnaky A., Gill H., Newman L.P., Danaher J. (2022). Australians’ Experience, Barriers and Willingness towards Consuming Edible Insects as an Emerging Protein Source. Appetite.

[31] Lucchese-Cheung T., Aguiar L.K.D., Da Silva R.F.F., Pereira M.W. (2020). Determinants of the Intention to Consume Edible Insects in Brazil. J. Food Prod. Mark..

[32] Liu A.-J., Li J., Gómez M.I. (2019). Factors Influencing Consumption of Edible Insects for Chinese Consumers. Insects.

[33] Guiné R.P.F., Duarte J., Chuck-Hernández C., Boustani N.M., Djekic I., Bartkiene E., Sarić M.M., Papageorgiou M., Korzeniowska M., Combarros-Fuertes P. (2023). Validation of the Scale Knowledge and Perceptions about Edible Insects through Structural Equation Modelling. Sustainability.

[34] Likert R. (1932). A Technique for the Measurement of Attitudes. Arch. Psychol..

[35] Broen M.P.G., Moonen A.J.H., Kuijf M.L., Dujardin K., Marsh L., Richard I.H., Starkstein S.E., Martinez–Martin P., Leentjens A.F.G. (2015). Factor Analysis of the Hamilton Depression Rating Scale in Parkinson’s Disease. Park. Relat. Disord..

[36] Kaiser H.F., Rice J. (1974). Little Jiffy, Mark Iv. Educ. Psychol. Meas..

[37] Stevens J.P. (2009). Applied Multivariate Statistics for the Social Sciences.

[38] Rohm A.J., Swaminathan V. (2004). A Typology of Online Shoppers Based on Shopping Motivations. J. Bus. Res..

[39] Tanaka K., Akechi T., Okuyama T., Nishiwaki Y., Uchitomi Y. (2000). Development and Validation of the Cancer Dyspnoea Scale: A Multidimensional, Brief, Self-Rating Scale. Br. J. Cancer.

[40] Hair J.F.H., Black W.C., Babin B.J., Anderson R.E. (2009). Multivariate Data Analysis.

[41] Maroco J., Garcia-Marques T. (2006). Qual a fiabilidade do alfa de Cronbach? Questões antigas e soluções modernas?. Laboratório De Psicol..

[42] Davis F.B. (1964). Educational Measurements Their Interpretation.

[43] Erhard A.L., Águas Silva M., Damsbo-Svendsen M., Menadeva Karpantschof B.-E., Sørensen H., Bom Frøst M. (2023). Acceptance of Insect Foods among Danish Children: Effects of Information Provision, Food Neophobia, Disgust Sensitivity, and Species on Willingness to Try. Food Qual. Prefer..

[44] White K.P., Al-Shawaf L., Lewis D.M.G., Wehbe Y.S. (2023). Food Neophobia and Disgust, but Not Hunger, Predict Willingness to Eat Insect Protein. Personal. Individ. Differ..

[45] Escalante-Aburto A., Rodríguez-Sifuentes L., Ozuna C., Mariscal-Moreno R.M., Mulík S., Guiné R., Chuck-Hernández C. (2022). Consumer Perception of Insects as Food: Mexico as an Example of the Importance of Studying Socio-Economic and Geographical Differences for Decision-Making in Food Development. Int. J. Food Sci. Technol..

[46] Gómez-Corona C., Valentin D. (2023). The Crispy Cricket—Attitudes, Habits, and Tradition in Insect Consumption. Food Qual. Prefer..

[47] Florença S.G., Correia P.M.R., Costa C.A., Guiné R.P.F. (2021). Edible Insects: Preliminary Study about Perceptions, Attitudes, and Knowledge on a Sample of Portuguese Citizens. Foods.

[48] Dupont J., Fiebelkorn F. (2020). Attitudes and Acceptance of Young People toward the Consumption of Insects and Cultured Meat in Germany. Food Qual. Prefer..

[49] Gahukar R.T. (2020). Edible Insects Collected from Forests for Family Livelihood and Wellness of Rural Communities: A Review. Glob. Food Secur..

[50] Nowak V., Persijn D., Rittenschober D., Charrondiere U.R. (2016). Review of Food Composition Data for Edible Insects. Food Chem..

[51] Payne C.L.R., Scarborough P., Rayner M., Nonaka K. (2016). A Systematic Review of Nutrient Composition Data Available for Twelve Commercially Available Edible Insects, and Comparison with Reference Values. Trends Food Sci. Technol..

[52] Lu M., Zhu C., Smetana S., Zhao M., Zhang H., Zhang F., Du Y. (2024). Minerals in Edible Insects: A Review of Content and Potential for Sustainable Sourcing. Food Sci. Hum. Wellness.

[53] Kunatsa Y., Chidewe C., Zvidzai C.J. (2020). Phytochemical and Anti-Nutrient Composite from Selected Marginalised Zimbabwean Edible Insects and Vegetables. J. Agric. Food Res..

[54] Zhang E., Ji X., Ouyang F., Lei Y., Deng S., Rong H., Deng X., Shen H. (2023). A Minireview of the Medicinal and Edible Insects from the Traditional Chinese Medicine (TCM). Front. Pharmacol..

[55] Siddiqui S.A., Li C., Aidoo O.F., Fernando I., Haddad M.A., Pereira J.A.M., Blinov A., Golik A., Câmara J.S. (2023). Unravelling the Potential of Insects for Medicinal Purposes—A Comprehensive Review. Heliyon.

[56] Figueiredo R.E.C.R., Vasconcellos A., Policarpo I.S., Alves R.R.N. (2015). Edible and Medicinal Termites: A Global Overview. J. Ethnobiol. Ethnomed..

[57] Tanga C.M., Ekesi S. (2024). Dietary and Therapeutic Benefits of Edible Insects: A Global Perspective. Annu. Rev. Entomol..

[58] Hoon Lee J., Kim Y.-J., Kim T.-K., Song K.-M., Choi Y.-S. (2024). Effect of Ethanol Treatment on the Structural, Techno-Functional, and Antioxidant Properties of Edible Insect Protein Obtained from *Tenebrio Molitor* Larvae. Food Chem..

[59] Lee J.H., Kim T.-K., Kim Y.J., Kang M.-C., Song K.-M., Kim B.-K., Choi Y.-S. (2023). Structural, Physicochemical, and Immune-Enhancing Properties of Edible Insect Protein Isolates from *Protaetia Brevitarsis* Larvae. Food Chem. X.

[60] Orsi L., Voege L.L., Stranieri S. (2019). Eating Edible Insects as Sustainable Food? Exploring the Determinants of Consumer Acceptance in Germany. Food Res. Int..

[61] Gałęcki R., Sokół R. (2019). A Parasitological Evaluation of Edible Insects and Their Role in the Transmission of Parasitic Diseases to Humans and Animals. PLoS ONE.

[62] Murefu T.R., Macheka L., Musundire R., Manditsera F.A. (2019). Safety of Wild Harvested and Reared Edible Insects: A Review. Food Control.

[63] Imathiu S. (2020). Benefits and Food Safety Concerns Associated with Consumption of Edible Insects. NFS J..

[64] Zugravu C., Tarcea M., Nedelescu M., Nuţă D., Guiné R.P.F., Constantin C. (2023). Knowledge: A Factor for Acceptance of Insects as Food. Sustainability.

